# Transitioning to practice: a qualitative investigation of Australian graduate naturopath’s experiences of being in practice

**DOI:** 10.1186/s12906-021-03475-z

**Published:** 2021-12-15

**Authors:** Matthew J. Leach, Larisa A. J. Barnes, Andy McLintock, Helene M. Diezel, Kimberley Ryan, Amie E. Steel

**Affiliations:** 1 National Centre for Naturopathic Medicine, Southern Cross University, Military Road, East Lismore, Lismore, NSW 2480 Australia; 2 University Centre for Rural Health, University of Sydney, 61 Uralba Street, Lismore, NSW 2480 Australia; 3 Faculty of Health, University of Technology Sydney, Broadway Street, Ultimo, NSW 2007 Australia; 4 School of Social Science, University of Queensland, 280-284 Sir Fred Schonell Drive, St Lucia, QLD 4061 Australia; 5 Endeavour College of Natural Health – Office of Research, Wickham Street, Fortitude Valley, QLD 4006 Australia; 6 Australian Research Centre in Complementary and Integrative Medicine, Faculty of Health, University of Technology Sydney, Ultimo, NSW 2006 Australia

**Keywords:** Clinical practice, Experience, Graduate, Health services research, Naturopathy, Professional practice, Qualitative research, Transition

## Abstract

**Background:**

The transition from student to practitioner can be challenging, resulting in stress, burnout and attrition. While there has been ample research examining graduate medical and allied health practitioner experiences of transitioning to practice, there is a paucity of research exploring such experiences in newly qualified naturopathic medicine practitioners. In light of this knowledge gap, the objective of this study was to ascertain the experiences of practicing as a naturopath in Australia within the first 5 years post-graduation.

**Methods:**

Using a qualitative descriptive approach, recent graduates of an Australian Bachelor of Naturopathy (or equivalent) program were invited to participate in a semi-structured telephone interview to address the study objective. Data were analysed utilising a framework approach.

**Results:**

A total of 19 new graduates (94.7% female; 57.9% aged 40–59 years) undertook an interview. Five inter-related themes emerged from the data: practitioner, practice, proprietorship, professions, and perceptions. Connected with these themes were contrasting feelings, multiplicity of duties, small business challenges, professional collaboration, and professional identity, respectively.

**Conclusions:**

Participants were generally content with their decision to become a naturopath. However, most were confronted by a range of challenges as they transitioned from graduate to practitioner, for which many felt ill-prepared. In light of the complexity of the issue, and the potential impact on the sustainability of the profession, it is evident that a multi-pronged, multi-stakeholder approach would be needed to better support graduate naturopath transition to practice.

## Background

The health services sector represents one of the largest and fastest growing industries across the globe [1]. Despite the increasing demand for these services, workforce attrition remains a cause of concern [2, 3]. Myriad factors have been attributed to health workforce attrition, one of which is preparedness for practice [4].

The period of transition from undergraduate student to practitioner can be challenging for newly qualified health professionals as they encounter changes in responsibility, workload, workplace culture, patient/provider relationships and support [4–6]. Evidence indicates newly qualified health professionals often experience feelings of stress, anxiety, fatigue, uncertainty and insecurity during this transitional period, which can be heightened if students feel inadequately prepared for the realities of professional practice [4–6]. For some newly qualified health professionals, these feelings of ill-preparedness can lead to disillusionment with practice [7], which can in turn, amplify intentions to leave the profession [4].

Research involving graduate nurses, pharmacists, occupational therapists, medical doctors, radiographers, midwives and veterinarians has provided valuable insights into the experiences of these professional groups as they transition from undergraduate training to clinical practice [8–19]. These studies highlight the importance of professional identity [10, 14], support mechanisms [8, 15, 17, 18], clinical experience during undergraduate education [9, 11] and the development of non-technical attributes (e.g. communication, interrelationship and problem-solving skills) in easing the impact of the transition period on newly qualified health professionals [16]. Overall, study findings support the view that the burden of responsibility for practice preparedness should not be placed solely on students/ practitioners, but shared by all stakeholders, including education providers and healthcare providers [19].

While the experiences of transitioning from student to practitioner are similar across health disciplines, they are not identical. This suggests that there are nuances between disciplines in the challenges encountered during this transition period. So, for disciplines yet to explore graduate transition to practice – such as naturopathic medicine – it is premature to speculate what the transitional challenges may be and accordingly, how they could be addressed.

Research and discourse on the experiences of practicing as a newly qualified practitioner is lacking in the field of naturopathic medicine. To address this knowledge gap, we used a qualitative descriptive approach to gather rich information on recent graduate naturopaths’ experiences of practicing naturopathic medicine in Australia. Insights gained from this study are expected to have important implications for naturopathic medicine education and practice in Australia and abroad.

## Methods

### Design

Qualitative descriptive study [20]. All methods were carried out in accordance with relevant guidelines and regulations [21].

### Aim and objectives

The aim of the study was to describe the transition to practice for recently graduated naturopaths (i.e. graduated within the past 5 years) in Australia. The specific objectives were to (A) determine graduate naturopath’s perceptions of their preparedness for clinical practice, (B) explore graduate naturopath’s views of being in the naturopathic medicine profession, and (C) ascertain graduate naturopath’s experiences of practicing as a naturopath. This manuscript addresses the latter objective. Papers addressing the other two objectives are currently in development.

### Participants

Individuals graduating from an Australian Bachelor of Naturopathy (or equivalent) program within the last 5 years were eligible to participate. The five-year timeframe was consistent with definitions of ‘recent graduate’ reported in other large studies [22]. At the time of this study, there were four Bachelor of Naturopathy programs in Australia, which ranged in duration between 3 and 4 years. The curricula of these degrees focused heavily on the biomedical sciences, social sciences, clinical sciences, nutritional medicine, and herbal medicine [23]. Graduates not in clinical practice were excluded from the study.

A convenience sampling method was utilised, with the study promoted through naturopathic practitioner Facebook groups. Sample size was not determined a priori; instead, an adaptive approach was applied, whereby data collection was ceased once data saturation had been reached (i.e. no new information arose from the interviews) [24].

### Interview schedule

A semi-structured interview schedule was developed to address the study objectives. The schedule was informed by previous published research, the research team’s knowledge of the field, and the research objectives. The schedule was piloted with a convenience sample of five recent naturopathy graduates, with some questions requiring minor modification to improve clarity. The interview questions addressing study objective C are listed in Table 1.

**Table 1:** Interview questions addressing study objective C

∙ Could you describe how you felt on your first day in naturopathic medicine practice? ∙ Could you describe how you feel about being in naturopathic clinical practice at present? ∙ What was your experience of establishing your clinical practice? ∙ How would you describe your current clinical practice arrangement? ∙ How much of your naturopathic medicine clinic work time is spent with patients? How much is spent doing other clinic-related tasks? What sort of tasks are they? ∙ Could you describe your experience of being a practitioner in the broader naturopathic medicine profession? (e.g. is it collegial or competitive?)	

### Procedures

Individuals expressing an interest in the study (either by email or telephone), were emailed a detailed participant information sheet. The information sheet outlined the purpose of the study, participant eligibility criteria, and the rights and expectations of participants. Interested individuals meeting the eligibility criteria were invited to sign and return the written consent form. On receipt of the consent form, a member of the research team contacted the consenting participant to arrange a mutually suitable time to conduct the telephone interview. The interviews (which were conducted by an experienced interviewer [KR]) were scheduled for 60 min, and were audio-recorded using a voice recorder. Audio-recordings were transcribed verbatim by an external transcription service, and were anonymised using unique alphanumerical identifiers.

### Data analysis

Interview transcripts were cross-checked against audio-recordings (by KR) to verify the accuracy of transcripts. The transcripts were subsequently uploaded into NVivo10 (QSR International, Melbourne, Australia) for data analysis. Data were analysed using an inductive framework approach [25]. In brief, the framework approach comprised five steps: (i) familiarisation with data; (ii) development of a thematic framework; (iii) indexing (i.e. assigning blocks of data to particular themes); (iv) charting (i.e. assigning specific data to specific themes and subthemes); and (v) mapping and interpretation (i.e. defining key phenomena, identifying associations, offering explanations) [25]. The primary analysis was conducted by one researcher (HD), and triangulated by a second researcher (ML). Divergent interpretations were resolved by discussion.

## Results

### Description of participants

Nineteen graduate naturopaths undertook a telephone interview. The duration of the interviews ranged between 50 and 80 min, with a mean duration of 60 min. Participants were predominantly female (95%), aged 40–49 years (32%) and resided in Queensland (58%) (Table 2). More than one-half of participants worked as a casual employee (63.2%) within a multimodality clinic (57.9%) in an urban location (63.2%). All participants graduated with a Bachelor of Naturopathy degree within the past 5 years, with two-thirds graduating in the last 2 years (63.2%). Correspondingly, the majority of participants had been in clinical practice as a naturopath for less than 2 years (73.7%, 14/19).

**Table 2:** Characteristics of participants (*n* = 19)

Characteristic	n (%)
**Age**	
20–29 years	4 (21.1)
30–39 years	4 (21.1)
40–49 years	6 (31.6)
50–59 years	5 (26.3)
**Sex**	
Female	18 (94.7)
Male	1 (5.3)
**Practice location – state/territory**	
Queensland	11 (57.9)
Victoria	3 (15.8)
New South Wales	2 (10.5)
South Australia	2 (10.5)
Western Australia	1 (5.3)
Tasmania	0 (0.0)
Australian Capital Territory	0 (0.0)
Northern Territory	0 (0.0)
**Practice location - regionality**	
Urban	12 (63.2)
Rural	4 (21.1)
Not specified	3 (15.8)
**Practice setting**^**a**^	
Multi-modality clinic	11 (57.9)
In-home practice	8 (42.1)
In-store room or space	5 (26.3)
House calls	1 (5.3)
**Employment status**	
Casual employment	12 (63.2)
Part-time employment	5 (26.3)
Full-time employment	2 (10.5)
**Time elapsed since graduating from naturopathy degree**	
1 year	9 (47.4)
2 years	3 (15.8)
3 years	3 (15.8)
4 years	1 (5.3)
5 years	3 (15.8)
**Period of time in naturopathic clinical practice**	
Less than 12 months	10 (52.6)
12–23 months	4 (21.1)
24–35 months	3 (15.8)
36–47 months	1 (5.3)
48–59 months	1 (5.3)

^**a**^Multiple response options could be selected

### Thematic framework

**Fig. 1:**
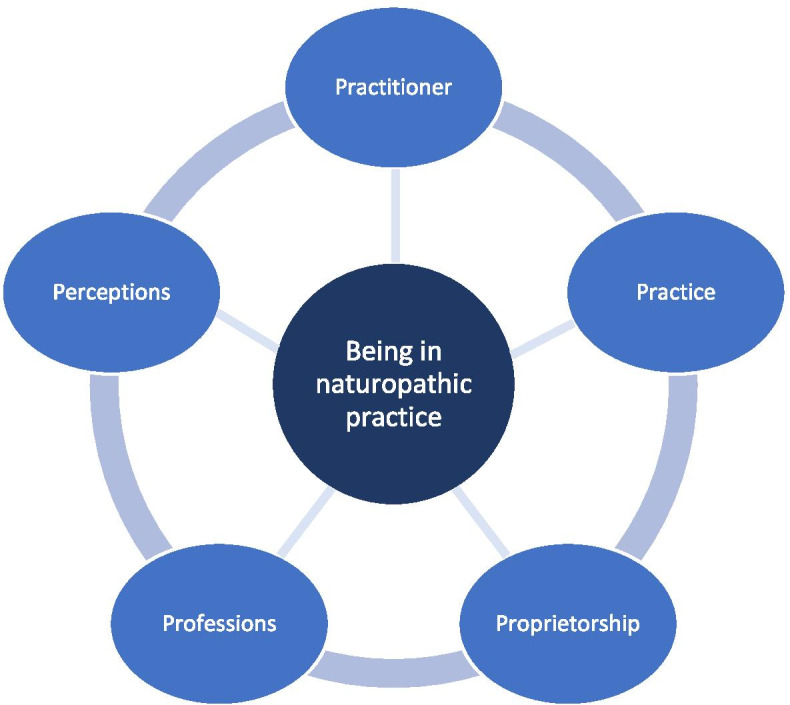
‘Being in naturopathic practice’ – a thematic framework

Five themes emerged from the data, which together formed the thematic framework of the study (Fig. 1). These themes were: (1) Practitioner – contrasting feelings, (2) Practice – multiplicity of duties, (3) Proprietorship – small business challenges, (4) Professions - professional collaboration, and (5) Perceptions – professional identity. Each of these themes, and their respective subthemes (where applicable), are unpacked below.

### Practitioner – contrasting feelings

Many participants expressed feelings of ambivalence when practicing as a graduate naturopath, with feelings oscillating between excitement and trepidation. These mixed feelings were particularly pronounced when graduate naturopaths were asked to comment on their first day of practice, as one participated articulated:

*I guess excited and terrified equally at the same time*. (Participant #1)

For many graduate naturopaths, these feelings of anxiety continued for an extended period of time:

*There was a lot of anxiety there and that took a little bit of time to get through it and I'm sure it's like that with a lot of people.* (Participant #3)

*So, there’s been a lot of emotion, and it’s been incredible to say the least the amount of stress I’ve been under.* (Participant #17)

Self-doubt and a perceived lack of confidence emerged as possible explanations for the anxiety experienced by graduate naturopaths, as two participants summarised:

*In reality, twenty percent confidence and eighty percent doubt…* (Participant #7)

*…it’s been a really long process to grow into being confident to be able to do what I wanted to do. I’m still growing and finding there’s still so much to learn and do.* (Participant #12)

Other participants found the transition from student (in a supported learning environment) to graduate naturopath (in private practice) somewhat unsettling as isolation crept in and support dwindled:

*I find being the only naturopath there very isolating, and until I came on board with the second practice a couple of weeks ago,…I felt pretty overwhelmed making all those decisions by my own and not having anybody to bounce things off on; so that was pretty challenging for the first four or five months.* (Participant #1)

Conversely, other graduate naturopaths relished the opportunity to be in clinical practice, reporting the transition to practice as gratifying, as exemplified by one participant:

*I feel privileged to be here and I feel humbled every time a new patient walks through my door. I really do.* (Participant #10)

For several graduates, being in clinical practice helped validate their decision to become a naturopath, as was eloquently stated by two participants:

*When I am in…a consult, I love it, it’s exactly where I want to be*. (Participant #7)

*I’m happy! I'm happy! I'm grateful every day that I decided to become a naturopath…I can't come back to do any other work. Only this. This is my goal for all my life, for future life.* (Participant #14)

Practicing as a graduate naturopath was rewarding in other ways as well, with some participants cherishing the opportunity to apply and build-on the knowledge acquired during their undergraduate degree to clinical practice:

*My naturopathic knowledge is starting to really grow now. I'm five years out now and that naturopathic knowledge, my knowledge of herbs and knowledge of nutrients and knowledge of product[s] is growing, growing, growing...* (Participant #10)

Other graduate naturopaths were pleased, and somewhat reassured, to finally see the knowledge and skills developed during their degree having a positive impact on people’s lives:

*I think every client so far has come back at least once so I feel good about that…which means that it makes me feel more confident in myself that I’m doing something good.* (Participant #18)

### Practice – multiplicity of duties

The duties performed by graduate naturopaths were diverse, with activities broadly categorised into direct client care, indirect client care, and non-clinical duties. In terms of direct client care (i.e. consulting with the client), graduate naturopaths generally spent between one and one-and-a-half hours delivering initial consultations, and anywhere between half-an-hour and an hour for follow-up consultations. The few participants that described the nature of these consultations had mentioned using this time for “testing” (Participant #1), “preparing herbals” (Participant #2), “ask[ing]…questions” and completing physical assessments (Participant #4).

Most graduate naturopaths’ workloads were spent undertaking indirect client care activities (i.e. actions performed outside the clinical consultation). These activities were performed prior to and/or following the clinical consultation, and ranged in duration from 10 to 30 min, to 1–2 h, to more than 3 h per client. For many graduate naturopaths, this time was used to research the client case/treatment options, prepare client treatment plans, prepare emails/letters for clients and other health providers, finalise paperwork, review pathology results, and/or prepare/order treatments. These broad range of activities are exemplified in the following comment:

*…before a client comes in,…I might spend a couple of hours just researching what might be going to happen, and then if I’ve seen that patient once already, then I'm researching what it is I'm giving them. I might be making them up compounded nutrients…keep[ing] my patients notes up-to-date…reviewing pathology results…* (Participant #10)

Graduate naturopaths also spent time on activities not directly related to client care. These duties included marketing (including website management, blog writing and social media posting), book-keeping, stock control, and professional development. These activities required between 1 and 10 h of a graduate naturopath’s time per week. The fraction of time allocated to these non-clinical duties was articulated by the following participant:

*Sixty [percent of my clinic time] is like non-patient work…like social media accounts…book work…[the] website… patient management software…intake forms and those sorts of things.* (Participant #19)

### Proprietorship – small business challenges

Establishing a small business / clinical naturopathic practice was challenging, and at times overwhelming, for many graduate naturopaths. Business administration was an area several graduate naturopaths felt inadequately prepared for, as on participant indicated:

*I expected the transition into naturopathy [to be] a little bit easier, but I didn’t expect to have to do so much on the business side of the business in the non-naturopathic things. I didn’t expect that to be so huge.* (Participant #12)

Another challenge graduate naturopaths faced when setting up a clinical naturopathic practice was building a client base:

*Building up clients has been slower than I optimistically thought that it was going to be…* (Participant #1)

*I wasn't prepared in terms of how hard it would be to actually get clients.* (Participant #2)

The slow client inflow, and high business overheads, meant many graduate naturopaths struggled financially to establish a clinical naturopathic practice:

*I started out working basically out of my car just doing house calls because I couldn't afford to hire a room or anything like that.* (Participant #3)

*my first year…I wrote off five to eight grand in loss and that was so sobering; it was like OMG this business is actually haemorrhaging me money…* (Participant #16)

Financial difficulties caused several graduate naturopaths to question the viability of naturopathy as a career, as one participant summarised:

*I've just got a new job, so currently it would be like 70% of my other job supports me, and this one [naturopathy] is basically my hobby.* (Participant #2)

### Professions - professional collaboration

An issue of importance to graduate naturopaths was establishing networks and collaborations with providers both within (i.e. intraprofessional collaboration) and outside (i.e. interprofessional collaboration) the naturopathic profession. In terms of intraprofessional collaboration, participants indicated that they felt supported by the profession, as was epitomised in the following quote:

*At the moment, I'm definitely feeling that it's [naturopathy] very supportive and very collaborative.* (Participant #5)

Support was afforded through social media networks, clinic colleagues, fellow graduate naturopaths and past supervisors and lecturers:

*Facebook groups has been the biggest support…Just seeing what ideas other people were coming up with and networking.* (Participant #3)

*…you've got so much support and you've got so many - you've got all your colleagues around, you've got your supervisors, your lecturers.* (Participant #11)

*And it was nice to have another...’it’ being a clinic with another naturopath, because after seeing a client or when I finished for the day we always have a little chat, or like a de-brief. It's really nice.* (Participant #19)

Some graduate naturopaths also obtained support through a more formal mentorship arrangement:

*I'm paying someone to mentor me as well, so I have good support which is really useful…I think it would be lonely and really tricky without that.* (Participant #19)

Notwithstanding, there was a group of graduate naturopaths who were concerned by the perceived paucity of mentorship opportunities in naturopathy, as one participant articulated:

*There’s no specific mentoring program, or website, or anything like that, that you can go on as a new up and coming naturopath…* (Participant #4)

Similarly, several graduate naturopaths expressed there was not enough collaboration in naturopathy, going so far as to say that the profession was somewhat cut-throat:

*I've actually surprisingly found it to be quite competitive and ego-centric….as a whole it could be a little bit more collegial.* (Participant #2)

Graduate naturopaths also shared experiences regarding the importance of collaborating with professionals outside the discipline of naturopathy, including other complementary medicine providers (e.g. osteopaths, chiropractors), allied health providers (e.g. psychologists, physiotherapists) and general practitioners. Within this context, participants highlighted the value of interprofessional referral, as participant 5 underscored:

*There's got to be referrals because…we're not qualified to do everything, so I think there's got to be that collaboration.* (Participant #5)

For these naturopaths, referring to other professions was largely a positive experience:

*Yeah, I do that [refer] all the time…[to mostly osteopaths and doctors]…And generally I get pretty good feedback.* (Participant #6)

*My local doctor and I have a good relationship and she refers to me…[and] I’d refer through to her.* (Participant #11)

However, other graduate naturopaths had encountered some difficulties in trying to develop interprofessional collaborations. One of these challenges was the hostility and/or lack of engagement from some professions:

*And I try to let doctors know that I'm happy to support their patients and that l want to be collaborative. But getting a response is not always easy and getting collaboration is not always easy…So you don't get any communication at all or you get a complete put down where they'll tell the patient, “well, you can go and see the naturopath, but nothing she gives you is going to make you feel better.”* (Participant #10)

Another challenge, as reported by one graduate naturopath, was the inadequate training provided during their degree on preparing referrals:

*I never write a letter. In fact, that would be one thing that I recommend that the course taught more; making writing letters become like second nature. We did, but it wasn't enough for me to feel comfortable doing on my own.* (Participant #9)

### Perceptions – professional identity

Graduate naturopaths felt confronted by the level of uncertainty and misperception among patients regarding the definition and role of naturopathy, as eloquently stated by one participant:

*Something that surprised me when I graduated and left the bubble of [educational institution] was how many people didn’t really know what a naturopath was or did.* (Participant #1)

The public uncertainty of naturopathy also extended to the tools of the trade:

*It’s a challenge. They [patients] don’t understand what it means to use herbs, how does it work, how supplementation works.* (Participant #14)

Graduate naturopaths discussed the lack of unity among professional associations and the lack of statutory regulation contributed to the profession’s struggling identity, advancing that addressing these issues would help improve the visibility of naturopathy in Australia:

*there’s a bit of a division in our profession on that issue [registration] and that might be holding us back a little bit. I’m just going to finish by saying maybe that issue comes down to unity within our profession.* (Participant #1)

*I'm really hoping that that [professional visibility] would change in the future…if all these associations came together and worked together…[to]…promote the profession. Otherwise that’s not going to happen.* (Participant #3)

*It’s [naturopath] not a protected title and therefore you can have someone who has done a weekend course or online course…[or]… has studied for four years fulltime and has a bachelor…so…I don’t know that the public are aware of the differences when you are just given a title.* (Participant #8)

## Discussion

The transition from graduate to practitioner has been examined across many health disciplines [13, 15, 26, 27], yet has received little attention in the profession of naturopathic medicine. In response to this knowledge gap, this qualitative study explored for the first time, the complexities of ‘being in naturopathic practice’, from the vantage point of recently qualified Australian naturopaths. These complexities connected to one of five interrelated themes: Perceptions, Professions, Practitioner, Practice and Proprietorship.

The transition from student to practitioner is a complex, but achievable, socialisation process whereby “practitioners are inducted, trained and credentialled into the culture of their profession and incorporate the profession’s values and norms into their self-concept” [28]. In other words, as students move from a supported educational environment to being independent, credentialed clinicians, they begin to develop their identity as a practitioner [13, 28, 29]. To some extent, this identity emerges as practitioners begin to master professional competencies. While development of these competencies often begins in the education setting [29, 30], the assimilation of personal and professional values and behaviours typically occurs after graduation, when the graduate is in clinical practice [30, 31]. This was certainly the case for participants in this study, where professional identity emerged as an issue of concern after being in naturopathic practice.

As participants transitioned into clinical practice, they were confronted by a perceived lack of unity among professional associations. In Australia, there are numerous professional associations for which a naturopath can become a member. These professional associations are self-regulatory organisations that primarily serve to establish and oversee professional conduct and education standards [32, 33]. However, this diversity – and associated division – has been identified previously as negatively impacting naturopathic education [34] and practice [35] in Australia. While it is expected that professional communities also should play a role in improving recognition amongst members, identifying commonalities in work practices, procedures and patient interactions, fostering positive intra-professional relations, and reducing excessive intra-professional competition [36], participants did not feel that professional associations effectively fulfilled these duties as yet.

Participants identified statutory regulation as a possible solution to fostering professional unity and improving professional identity. While there is no statutory regulatory authority that currently governs naturopathic medicine in Australia, there have been moves towards regulating the profession [33] and to coordinating activities among naturopathic professional bodies supportive of statutory registration. Nonetheless, as naturopathy currently remains self-regulated (at least in Australia), perceptions of fragmentation and disunity amongst the naturopathic profession are likely to persist – not just within the profession, but also outside the profession.

Participants were not prepared for the level of uncertainty and misperception among patients regarding the definition and role of naturopathy, and the tools of the trade. Of concern is the negative public perceptions of the profession, as this can contribute to practitioner work dissatisfaction, potentially impacting workforce performance and retention [37–39]. This raises questions regarding who is responsible for promoting and advocating the naturopathic profession, and what can be done to enact these responsibilities. Answers to these questions may be an impetus to fostering improvements in the professional identity of naturopathic medicine practitioners.

Public perceptions of a profession can be compounded when medical doctors question the role of a profession in patient care [37–39]. Indeed, participants in this study expressed difficulties in trying to establish interprofessional collaborations, particularly with medical practitioners. A phenomenon, unfortunately, also reported by other allied health and complementary medicine practitioners [37–40], suggesting that this experience is not unique to Australian naturopaths. Nevertheless, if recommendations for improving interprofessional communication and care between medical doctors and naturopaths were followed [40], this may address some of these challenges to being in naturopathic practice – particularly those related to ‘perceptions’ and ‘professional collaboration’. Given the recognised importance of interprofessional collaboration in health care [41], particularly in chronic disease populations commonly accessing naturopathic care [42], these challenges should not go unaddressed.

A related issue impacting participant transition to practice was intraprofessional collaboration, namely the lack of quality mentorship opportunities for novice naturopathic medicine practitioners. Formal mentorship has been found to be an integral element of successful transition from student to practitioner in many health disciplines, including occupational therapy [12], nursing [15], radiography [13] and medicine [43]. Moreover, limited exposure to mentorship is shown to be associated with an increased risk of isolation, lack of confidence and feelings of stress in new health graduates [15, 39]. While mentorship has been identified as a key need for naturopathic researchers [44], it is unclear as to what form, style and amount of mentorship would positively impact transition to practice in new naturopathic medicine graduates. This knowledge gap warrants further exploration to determine how mentorship might best serve the needs of novice naturopathic medicine practitioners.

The experiences described by participating naturopathic graduates as they transitioned into practice were similar to those reported by other complementary, allied and biomedical practitioners [13, 15, 26, 27]. Although several participants in this study expressed concern, anxiety and self-doubt about embarking on professional practice, low levels of professional confidence are not uncommon in novice health practitioners, with levels often increasing as additional clinical experience is gained [12, 13, 15, 43, 45]. Furthermore, as professional confidence and experience increases, the practitioner’s clinical focus ordinarily shifts from technical competencies (i.e. developing specific clinical skills) to general competencies (i.e. mastering effective communication, problem solving, social responsibility) [43]. In doing so, the practitioner begins to develop greater certainty about their professional identity and role [46]. As has been demonstrated in occupational therapists, the development of these attributes may be accelerated through mentorship, earlier clinical experiences, and improvements in the teaching-practice nexus [26], which to some extent shifts a large onus of the responsibility for developing practitioner confidence on education providers [47].

As most graduate naturopaths in Australia work in private practice as solo practitioners [48, 49], a multiplicity of proprietorship and non-clinical duties are often undertaken alongside clinical responsibilities [50]. Unsurprisingly, the burden of non-clinical duties added to the challenges study participants experienced during their transition to practice. This finding is not exclusive to naturopathic graduates, with nutritionists and herbal medicine practitioners in New Zealand reporting a comparable distribution of clinical and non-clinical tasks [38]. Additionally, study participants expressed concern about being insufficiently prepared to manage a business – a sentiment shared by Australian complementary medicine practitioners more broadly [51] as well as nutrition therapists in the United Kingdom [37]. Based on the experiences of participants in this study, it is possible that the development of adequate and authentic business administration skills within naturopathic medicine programs may better prepare graduates for clinical practice, and in turn, help mitigate the disquietude associated with graduate naturopathic transition to practice.

This study has a number of strengths and limitations. The use of Facebook to quickly and effectively facilitate participant recruitment is known to be a cost-effective, efficient method for targeted recruitment [52, 53]. Arguably, recruitment through Facebook enabled the researchers to reach naturopaths that had graduated from a variety of educational institutions, across Australia, which was not possible using conventional recruitment strategies. Biases inherent in the use of online recruitment (e.g. the exclusion of participants who may not have access to the Internet) [54] are limitations of this study. Additionally, convenience sampling and participant self-selection, commonly used in exploratory studies such as this one [55], also may be limitations to this study as only naturopaths who were in naturopathic practitioner Facebook groups were targeted. Using a self-selected, convenience sample limits the generalisability of the participants’ observations and experiences. However, qualitative research aims to deeply explore a phenomenon, in this case the views, beliefs and experiences of recent naturopathic graduates, in order to uncover and develop a deeper understanding of their transition to practice; an understanding that would not be possible to obtain from quantitative methods using ‘representative’ samples [56]. Additionally, the inclusion of participants across a 20 year age range, from five different Australian states, both rural and urban regions, and a variety of practice settings, strengthens the findings of this study.

## Conclusions

This research identified five interrelated themes regarding transition to practice, all centring around the construct of ‘being in naturopathic practice’. While participants were generally content with their decision to become a naturopath, most were confronted by a range of challenges as they shifted from graduate to practitioner, for which many felt ill-prepared. These challenges were encountered at various levels (from practitioner, to practice, patient, and profession), and contributed to feelings of stress, isolation and doubt about the viability of naturopathy as a career. As these issues threaten the sustainability of the profession and the wellbeing of its practitioners, addressing these issues should be considered a priority. In light of the complexity of the issue, it is evident that a multi-pronged approach would be needed to support graduate naturopath transition to practice; an approach of which all stakeholders of the profession should claim responsibility (including practitioners, educational institutions, professional associations, and regulators).

## Data Availability

The datasets used and/or analysed during the current study are available from the corresponding author on reasonable request.
